# Fine wine or sour grapes? A systematic review and meta-analysis of the impact of red wine polyphenols on vascular health

**DOI:** 10.1007/s00394-020-02247-8

**Published:** 2020-04-17

**Authors:** Samuel R. Weaver, Catarina Rendeiro, Helen M. McGettrick, Andrew Philp, Samuel J. E. Lucas

**Affiliations:** 1 School of Sport, Exercise and Rehabilitation Sciences, College of Life and Environmental Sciences, University of Birmingham, Birmingham, B15 2TT UK; 2 Centre for Human Brain Health, University of Birmingham, Birmingham, B15 2TT UK; 3 Institute of Inflammation and Ageing, College of Medical and Dental Sciences, University of Birmingham, Birmingham, B15 2WB UK; 4 Garvan Institute of Medical Research, Darlinghurst, NSW 2010 Australia; 5 St Vincent’s Clinical School, UNSW Medicine, UNSW Sydney, Sydney, NSW 2010 Australia

**Keywords:** Red wine, Polyphenols, Resveratrol, Vascular health, Blood pressure

## Abstract

**Purpose:**

Red wine polyphenols (RWP) are plant-based molecules that have been extensively studied in relation to their protective effects on vascular health in both animals and humans. The aim of this review was to quantify and compare the efficacy of RWP and pure resveratrol on outcomes measures of vascular health and function in both animals and humans.

**Methods:**

Comprehensive database searches were carried out through PubMed, Web of Science and OVID for randomised, placebo-controlled studies in both animals and humans. Meta-analyses were carried out on acute and chronic studies of RWP in humans, alongside sub-group analysis where possible. Risk-of-bias assessment was carried out for all included studies based on randomisation, allocation, blinding, outcome data reporting, and other biases.

**Results:**

48 animal and 37 human studies were included in data extraction following screening. Significant improvements in measures of blood pressure and vascular function following RWP were seen in 84% and 100% of animal studies, respectively. Human studies indicated significant improvements in systolic blood pressure overall (− 2.6 mmHg, 95% CI: [− 4.8, − 0.4]), with a greater improvement in pure-resveratrol studies alone (− 3.7 mmHg, 95% CI: [− 7.3, − 0.0]). No significant effects of RWP were seen in diastolic blood pressure or flow-mediated dilation (FMD) of the brachial artery.

**Conclusion:**

RWP have the potential to improve vascular health in at risk human populations, particularly in regard to lowering systolic blood pressure; however, such benefits are not as prevalent as those observed in animal models.

## Introduction

Red wine polyphenols (RWP) first came to prominence within the field of nutrition in the 1980′s with the identification of the “French Paradox” [1]. This theory pointed to the high levels of red wine consumed by the French as one factor that may explain the relatively low level of coronary heart disease (CHD) within the country, despite their high fat dietary habits [1]. Following on from these initial findings, accumulating evidence in the last 30 years suggests that specific polyphenol components within RWP can exert protective actions within the vascular system in both humans and animal models [2, 3]. In rodents, chronic supplementation with RWP and isolated RWP components has been shown to lead to improvements in both blood pressure and vascular responsiveness in excised vessels across a wide range of disease models, such as diabetes, obesity, hypertension and aging [4–10]. In vitro studies using human endothelial cells [11–13] also demonstrated reduced NADPH oxidase activity, reduced inflammation and increases in endothelial nitric oxide production via increases in endothelial nitric oxide synthase (eNOS) activity and intracellular Ca^2+^ concentration in response to RWP pre-treatment. Acute and chronic human randomized controlled trials have also revealed benefits of RWP supplementation within the vasculature, particularly, improvement in blood pressure and endothelial function [14, 15]. However, human intervention studies with RWP generally report less consistent findings across different populations (e.g., young, aged, obese, hypertensive, type II diabetic) and specific RWP [16–19]*,* in comparison to preclinical animal models.

RWP comprise a complex and varied array of molecules, including flavonoids such as ( +)-catechin, quercetin, anthocyanins, and the stilbene families of polyphenols [resveratrol (3, 5, 4′-trihydroxystilbene)] [2]. These have been isolated and studied with the objective of identifying the key polyphenolic components driving the beneficial effects on vascular health [2]. Evidence in vivo and under physiological relevant conditions in vitro, has shown that many of these compounds can have a wide range of physiological effects within the vasculature by activating key signal pathways such as insulin receptor 1 (IR-1) and sirtuin 1 (SIRT1), which are involved in insulin sensitivity, inflammation and cellular regulation [20]. Amongst RWP, resveratrol has emerged as a key component in regulating vascular homeostasis, and has been shown to interact with both IR-1 and SIRT1 when applied to either endothelial cells in culture and excised aortic arterial tissue ex vivo [21]. Modulation of these pathways has the potential to result in an increase in antioxidant capacity; improved metabolic health; and can act to regulate endothelial function through activation and upregulation of eNOS while inhibiting inflammatory pathways [21, 22]. Studies in rodents have shown that greater Nitric Oxide (NO) availability, reduced inflammation and improved antioxidant capacity can all contribute to improve vascular function through increased vasodilatory capacity, vessel compliance and reduced blood pressure [3, 23–25]. Indeed, animal studies in healthy subjects or disease models of hypertension, type II diabetes and metabolic syndrome, have shown improvements in blood pressure and vascular function following chronic supplementation with both whole extract polyphenols and pure compounds including resveratrol, quercetin and pterostilbene [6–8, 24, 26]. In contrast, in healthy human populations there is a consistent lack of efficacy on vascular outcomes [27, 28], but greater variability in the beneficial responses within clinical populations, including type II diabetes, obesity and hypertension [17, 19, 29–31].

There are a number of important aspects that must be considered when directly comparing polyphenol interventions in animal and humans. Firstly, the metabolic fate of dietary polyphenols is highly varied both within and between species: it can be dose-dependent, and be influenced by the background diet and composition of gut microbiota [32–35]. Further differences in metabolic rate between humans and animals demands that careful consideration is given to ensure that equivalent dietary relevant doses are administered across species [36]. Finally, animal studies benefit from highly controlled environments which include controlled background diets that are typically polyphenol-free. In humans this level of control is not possible, and in cases, where dietary restrictions are put into place adherence to restrictions, guidelines, and interventions themselves can vary greatly [37, 38].

Previous systematic reviews have mainly focussed on the effects of pure RWP in at-risk populations, with arterial blood pressure as the key outcome measure [14, 39, 40]. Limited attention has so far been given to effects of whole RWP, which are expected to be more relevant within the context of normal diets. Furthermore, the impact of these polyphenolic compounds on key predictive outcome measures of future cardiovascular risk, such as brachial flow mediated dilation (FMD)) as well as cerebral vascular function is limited. Finally, to the best of our knowledge, no review has assessed the consensus within animal model studies and directly compared these to the effects reported in humans. This systematic review, therefore, aims to quantify and compare the effects of whole RWP and pure resveratrol on whole body vascular health and function (blood pressure, flow-mediated dilation and CO_2_ reactivity in the cerebrovasculature) in both animal and human models. In addition, we aim to determine the impact of different human populations (e.g., health status, age and BMI) and study characteristics (e.g., type, dose and duration of supplementation) on vascular outcome measures in response to RWP.

## Methods

This review was carried out following the Preferred Reporting System for Systematic reviews and Meta-Analyses (PRISMA) guidelines. A full, updated version of the review protocol was produced following the PRISMA-P guide [41, 42] and published on the PROSPERO register (https://www.crd.york.ac.uk/prospero) under the registration number CRD42018103246, which including details on complete sample search query, inclusion and exclusion criteria, and data extraction and analysis.

### Search strategy

Systematic database searches were carried out from July to November 2018, through MEDLINE (PubMed, 1948 onwards), EMBASE (Ovid, 1980 onwards) and the Web of Science Core Collection (Clarivate Analytics, 1900 onwards). Search terms were selected based on the well supported PICO format, which separates terms based on Population, Intervention, Comparison and Outcome [41, 43] and are detailed in Table 1. Population terms were included to search for both animal model and human studies, to collect studies within both population types for comparison of the results of RWP supplementation in both, as previously stated. Full details of the search terms used alongside an example search for the Medline database can also be found on the PROSPERO register.

**Table 1:** Search terms used for database searches, based on the PICO System for search strategy development

Patient/population/problem	Intervention	Comparison/Control	Outcome
Mice Mouse Rodent* Animal Animal model[MH] OR Healthy adult* Young adult* Adult* Cardiovascular disease[MH] CVD TIIDM OR T2DM Diabet* Overweight Obese Elder* Old* adj3 adult* Aging	Red wine polyphenol RWP Resveratrol Pterostilbene[MH] *stilbene	Placebo Control Negative control (Polyphenol adj3 free adj3 control)	*Vascular function *Vascular responsiveness *Vascular reactivity Blood pressure (OR BP) Transcranial doppler (TCD) Flow mediated dilation (FMD) fMRI Blood flow Peripheral blood flow Cerebrovascular blood flow

### Selection criteria and screening procedure

Full citation results for all searches were collected and, following removal of duplicates, were screened for inclusion/exclusion using the Rayyan online screening tool [44]. Systematic reviews and meta-analyses found during literature searches were manually screened for additional studies that were not found in the results of initial database searches. Studies were initially screened by their abstracts and were included for full text screening and data extraction if: (1) a specific RWP supplement was orally administered, with doses stated and an appropriate placebo/control was administered; (2) outcome measures/methods included one or more measure of blood pressure; cerebral or peripheral vascular function, and (3) details of the specific population included health and disease status. This review aimed to determine the impact of extracted RWP and specific polyphenol isolates. Due to the differences in delivery, dosage and inter-variety/batch variability of whole wine/juice [45], all of which make direct comparison problematic, studies investigating the impact of unrefined grape juice or alcoholised/de-alcoholised red wine were excluded.

In relation to human-based studies, studies were included if the study design included appropriate randomisation, treatment blinding and either a placebo/control arm or a cross-over method was applied. In the case of animal-based studies, ex vivo analysis of vascular function in excised artery samples was also considered an acceptable outcome measure for inclusion.

Following initial screening, included studies were then assessed as full-texts to determine final inclusion/exclusion and were separated into human and animal study categories to ensure that full details were available regarding the supplement type and dosage; duration of supplementation; primary outcome measures; population demographics and clinical characteristics; and study design. Final exclusion produced a total of 89 studies for full data extraction; quality assessment and analysis; full details of the reasons for exclusion are shown in Table 2.

**Table 2:** Study exclusion and summary of reason/justification of exclusion from the present review

Number of studies	Study	Reason for Exclusion
6	Akaberi et al., 2016; Baile et al., 2011; Belcaro et al., 2013; Evans et al., 2016; Karatzi et al., 2009; Wong et al., 2013	Not RCT (review article or non-randomised/non-placebo control study)
19	Always et al., 2017; Bashmakov et al., 2014; Baur et al., 2006; Brasnyo et al., 2011; Care et al., 2016; Chan et al., 2008; Gliemann et al., 2013; Goh et al. 2014; Gordish et al., 2014; Robich et al., 2010; Palmisciano et l., 2015; Pollack et al., 2017; Poulsen et al., 2013; Shahraki et al., 2017; Soner et al., 2014; Thazhath et al., 2016;Tome-Carneiro et al., 2012a; 2012b; Zare et al., 2017	Primary outcomes for the review were not presented or not shown in full (e.g., no pre-trial data, no placebo results, only presented as figure)
17	Biesinger et al., 2016; Chan et al., 2008; Chu et al., 2011; Cruz, 2006; Dorri et al., 2017; Gordish et al., 2014; Idris-Khodja et al., 2013; Karatzi et al., 2004; Sarr et al., 2006; Silan et al., 2008; Subramanian et al., 2011; Taguchi et al., 2014; 2015; Toklu et al., 2010; Wang et al., 2002; Wang et al., 2005; Xu et al., 2009	Not oral administration of RWP or RSV (detail of supp not given/whole juice or wine/injection of supp/cell culture)
5	Bienholz et al., 2017; Chander et al., 2006; Lopez-Sepulveda et al., 2008; Mozafari et al., 2016; Song et al., 2005	No isolation of RWP effects (surgery response)
3	Botden et al., 2012; Wong et al., 2016; Xu et al., 2009	Duplicates

### Data extraction and quality assessment

A single reviewer (SW) completed the searches, study selection, data extraction and quality assessment. A second reviewer assessed all full-text exclusion justifications and was consulted in cases, where inclusion/exclusion was uncertain (CR). In the event of a disagreement between reviewers one and two, a third reviewer independently assessed the matter and made a final decision regarding inclusion/exclusion (SL). These precautions were taken to minimise the risk of single reviewer selection error, as recommended in the Cochrane guidelines [46].

Data extracted included publication details (authors, date, journal, title, etc.); study design characteristics (randomisation, placebo/controls, etc.); population details (number, sex, age, physical characteristics, health/disease status and medication usage); the type of RWP used; the dosage and duration used for supplementation; and all data available on pre- vs. post-supplementation and control vs. supplementation vascular measures. If data were not available for primary outcome measures reported in methods sections, the authors were contacted by the correspondence email address and the date of contact was recorded. In the case of studies that included multiple doses of a single supplement or comparisons of multiple supplements, all included measures were extracted and recorded as separate trials.

The quality of studies was judged in accordance with the Cochrane Collaborations recommendations with bias being judged based on six criteria covering random sequence generation; allocation concealment (selection bias); blinding of participants and researchers (performance bias); blinding of outcome assessment (detection bias); incomplete outcome data (attrition bias) and selective reporting (reporting bias); as well as the reporting of sources of funding and conflicts of interest (additional bias source) [47]. These sources of bias were rated as either high risk, uncertain risk or low risk, and allocated a score of 1, 0 or − 1, respectively, to give an overall rating of bias risk for each study. In accordance with well established guidelines regarding risk of bias assessment, these scores were not taken into account in determining the estimated effect size of each study, but were used to examine bias as a potential cause for heterogeneity within the results [43].

### Statistical analysis

#### Animal studies

Animal studies were evaluated to determine the proportion of studies that saw a significant improvement in blood pressure or vascular function following RWP treatment, including sub-group analysis between healthy and at-risk/disease-model populations. In addition, summary statistics were calculated to determine the range of supplements used and the average supplementation period, presented as mean and standard deviation or proportion of total studies included.

### Data extraction and synthesis

In human studies, the primary outcome measures were overall change in vascular measures between pre- and post-intervention, calculated as the difference in mean values. Blood pressure results were extracted as systolic and diastolic blood pressure, either as seated “office” measurement using a portable brachial artery cuff, or as ambulatory blood pressure over a 12- or 24-h period with the mean being included in subsequent quantitative analysis. Vascular functional measures of brachial artery dilation in response to shear stress were extracted either as percentage or absolute diameter change, as well as shear rate if reported (which uses diameter change and blood flow velocity to give an indication of artery wall shear stress). Transcranial doppler measures were extracted as cerebral blood flow velocity through the middle cerebral artery (MCAv) and/or posterior cerebral artery (PCAv), with comparison being made between studies using matching target artery velocity measures only.

If measures were presented as mean difference with 95% confidence intervals, the Cochrane Handbook method (7.7.7.2) for calculating standard difference from 95% CI’s was applied [48]. In the case of studies reporting non-parametric results in the form of median and interquartile range, the mean and standard deviation were estimated from the sample size, median and interquartile range [49]. Studies were separated based on whether the intervention was acute or chronic, and qualitative and quantitative analysis was carried out separately to differentiate the acute and chronic effects of supplementation. All data were calculated and presented as mean ± SD unless otherwise explicitly stated.

### Quantitative analysis

Mean differences between the start and end of the intervention period were calculated with comparison between group mean differences, and overall effect estimates were calculated by random effect models, and reported alongside estimate significance (*p* value) and heterogeneity (*I*^2^), with significance being determined based on a *p* value below an alpha value of *α* = 0.05. All blood pressure data were reported with the same unit of measure across all studies, allowing mean differences to be used for models to estimate a weighted mean difference. Vascular function data [e.g., flow-mediate dilation (FMD) response] were reported as both percentage change and diameter change; therefore, standardised mean differences were calculated to estimate overall effect size. The *I*^2^ statistic was examined to evaluate heterogeneity, with *I*^2^ > 50% and *I*^2^ > 75% indicative of substantial and considerable heterogeneity, respectively [50].

### Moderator and meta-regression analysis

Subgroup analyses were carried out to identify possible sources of heterogeneity; specifically by comparison of overall and subgroup estimated effects based on the type of supplement used and the health status of the participant cohort. Meta-regression was assessed using mixed effect models to evaluate the impact that potential moderators had on the estimated effect and heterogeneity of overall and subgroup effects. Models were run collectively to reduce the likelihood of failure to detect a moderator due to suppression, or over complication of moderator influence due to undetected confounding interactions between moderators [51]; with the exception of medication status, which was run in isolation on the at-risk subgroup due to the potential for interaction effects. The impact of moderators on the effects of supplementation was evaluated by assessing the proportion of heterogeneity each one accounted for, with significance determined by an Omnibus test for the overall model effect and Wald-type Chi-Squared tests for each moderator within the model.

### Sensitivity analysis

Externally standardised studentized deleted residuals were used to evaluate and identify potential outliers, based on the size of each study’s individual residual, with residual values < − 2 or > 2 considered to be outlying. The impact of outliers on the overall result was then assessed using model fit impact analysis (DFFITS and Cook’s distance); covariance from the mean; residual heterogeneity test statistics; overall result influence (hat values); and study weight [52]. If a study was found to be outlying and to have reasonable influence on the overall result, the random effect model was refitted and reported with and without influential outlying studies. All statistical analyses were performed in RStudio [53] using the Metafor meta-analysis package [54], and all effect estimates are reported as mean difference with 95% confidence intervals (MD, [95% CI]) unless otherwise stated.

### Search results

**Fig. 1:**
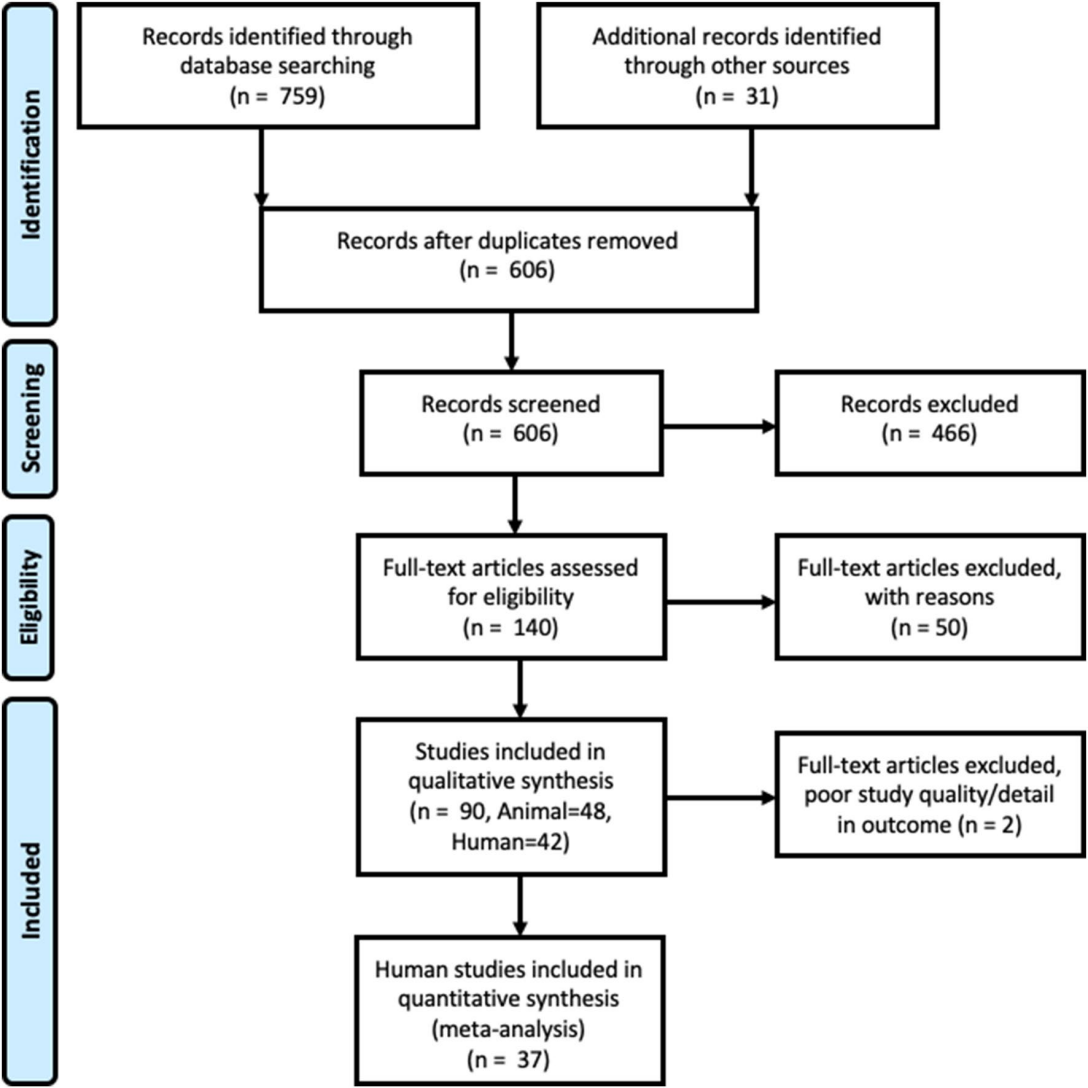
PRISMA flow diagram showing the search, screening and selection process, including eligible study numbers for both qualitative and quantitative synthesis

The search, screening and selection process for eligible studies is shown in Fig. 1. A total of 759 studies were found through database searches and were included in preliminary screening, with an additional 31 studies found in reviews that had been identified as relevant through the original database searches. Of these studies, following removal of duplicates a total of 607 were screened by title and abstract, 466 of these were excluded due to either a lack of randomisation of placebo/control group, usage of a supplement that did not match the inclusion criteria; or absence of details on the required primary outcome measures. 141 full-text articles were then screened and divided into animal and human studies, of which 50 were excluded from data extraction due to: incorrect study design/non-randomised control trials (RCT) study (*n* = 6); primary outcomes not present (*n* = 19); non-oral RWP supplementation (*n* = 17); RWP response not tested in isolation (*n* = 5); or data were a duplicate of an included study (*n* = 3).

## Results

### Animal studies

48 of the included studies were conducted in animal models, the majority of which were in rat models (*n* = 36) [4, 9, 55–87], alongside studies in mice (*n* = 9) [10, 88–95], rabbits (*n* = 2) [8, 96] and swine (*n* = 1) [7]. Of these all, but three, investigated the effects of RWP in clinical or disease models, including induced hypertension (*n* = 8), diabetes (*n* = 4), ageing (*n* = 6) and high fructose-induced vascular dysfunction (*n* = 7), with 25 studies including a healthy supplemented population (Table 3). A range of different RWP were used, including red wine extract, grape seed extract and whole-grape RWP, although the majority used single polyphenol supplementation with resveratrol (full details shown in Table 3). None of the studies investigated acute supplement effects alone, with the average duration of treatment being 8.5 ± 6.9 weeks. Significant improvements in vascular measures were seen after supplementation in 24% of studies in healthy animals, with specific significant effects on blood pressure (*n* = 21) and vascular function in arterial rings ex vivo (*n* = 10) in 24% and 40% of studies, respectively. Compared to response in healthy animals, studies in disease/clinical animal models saw significant improvements in the majority (88%) of studies, with 84% and 100% in blood pressure (BP) and vascular function measures, respectively.

**Table 3:** Study characteristics of animal intervention studies assessing the impact of red wine polyphenols on vascular health and function

Authors	Year	Animal	Disease/clinical model	Supplement	Dosage (mg/kg/day)	Duration	Measure	Healthy effect (sig)	Disease effect (sig)
Akar et al.	2011	Rabbit	T2D	Resveratrol	5 (mg/l)	(8–10 weeks)	EVVR		Yes
Akar et al.	2012	Rat	FVD	Resveratrol	50 (mg/l)	(10 weeks)	EVVR		Yes
Aribal-Kocatuerk et al.	2009	Rat	None	Resveratrol	20	(24 weeks)	SBP	Yes	
Aubin et al.	2008	Rat	HF	Resveratrol	20	(8 weeks)	SBP	No	Yes
Bernatova et al.	2002	Rat	L-NAME	Provinols	2.5	(1–3 weeks)	SBP and EVVR		Yes
Behbahani et al.	2016	Rat	SH	Resveratrol	40	(10 weeks)	EVVR and BP	No	Yes
Bhatt et al.	2011	Rat	SH	Resveratrol	5	(10 weeks)	SBP		Yes
Biala et al.	2010	Rat	Transgenic (Human Renin and angiotensin)	Resveratrol	800	(4 weeks)	SBP	No	Yes
Cheng et al.	2016	Rat	FVD	Resveratrol	10	(1 week)	BP		Yes
Cheng et al.	2013	Rat	FVD	Resveratrol	10	(2–4 weeks)	SBP and EVVR		Yes
Cheserek et al.	2016	Mouse	HFD	Resveratrol (and quercetin)	60 + 10 (g/kg chow)	(26 weeks)	EVVR		Yes
da Luz et al.	2011	Rat	None	Resveratrol	4 (mg/kg chow)	(4 weeks)	EVVR	No	
Dal-Ros et al.	2012	Rat	Ageing	RWP	100	(4 weeks)	BP and FMD		Yes
Dolinsky et al.	2013	Rat and Mouse	SH	Resveratrol	146 (rat) 320 (mice)	(5 weeks)	EVVR		Yes
Franco et al.	2013	Rat	Obese	Resveratrol	30	(30 days)	BP		Yes
Gendron et al.	2012	Mouse	Ageing	( +)-Catechin	0.75	(12–36 weeks)	EVVR	Yes	Yes
Gocmez et al.	2016	Rat	Ageing	Resveratrol	15	(2 weeks)	SBP and EVVR	No	Yes
Gordish et al.	2016	Rat	SH	Resveratrol	146	(4 weeks)	BP	No	Yes
Hort et al.	2012	Mouse	Transgenic (LDL Receptor)	RWE	3–30	(3 months)	BP		Yes
Inanaga et al.	2009	Mouse	AngII induced IL-6 expression	Resveratrol	10	(2 and 4 weeks)	BP	No	Yes
Jang et al.	2015	Rat	SH	GSE	15–30	(8 weeks)	SBP and EVVR		Yes
Jendekova et al.	2006	Rat	L-NAME	Provinols	40	(4 or 7 weeks)	SBP		Yes
Jiminez et al.	2007	Rat	SH	RWP	40	(5 weeks)	EVVR	No	Yes
Kavas et al.	2013	Rat	None	Resveratrol	20	(6 weeks)	BP	Yes	No
Khodja et al.	2012	Rat	Ageing	RWP	100	(2 or 4 weeks)	BP	No	Yes
Kosuru et al.	2018	Rat	FVD	Pterostilbene	20	(8 weeks)	EVVR	No	Yes
Lee et al.	2017	Rat	Spontaneous Heart Failure	Resveratrol or pterostilbene	2.5	(8 weeks)	SBP	No	Yes
Louis et al.	2012	Rat	Obese	Resveratrol	2.5	(4 weeks)	SBP	No	Yes
Majumdar et al.	2013	Rat	Ovariectomy-induced VD (+ tobacco extract)	Resveratrol	50	(8 weeks)	BP and CBF	No	Yes
Miatello et al.	2005	Rat	FVD	Resveratrol	10	(12 weeks)	SBP and EVVR	No	Yes
Mizutani et al.	2000	Rat	SH	Resveratrol	5	(8 weeks)	EVVR		Yes
Mizutani et al.	2001	Rat	SH	Resveratrol	1	(8 weeks)	SBP		No
Moraloglu et al.	2012	Rat	DOCA-Preclampsia	Resveratrol	20	(16 weeks)	SBP		No
Mozafari et al.	2015	Rat	T2D	Resveratrol	5–20	(4 weeks)	SBP and EVVR		Yes
Ozan et al.	2017	Rat	FVD	Resveratrol	10	(8 weeks)	BP and EVVR	No	Yes
Phyu et al.	2016	Rat	T2D	Resveratrol	2	(8 weeks)	SBP	Yes	Yes
Puzserova et al.	2006	Rat	Stressed	Provinols	20	(8 weeks)	SBP	Yes	No
Rezzani et al.	2009	Rat	Nephrotoxicity	Provinols	40	(3 weeks)	DBP and EVVR	No	Yes
Rivera et al.	2009	Rat	Obese	Resveratrol	10	(8 weeks)	SBP and EVVR	No	Yes
Robich et al.	2010	Swine	Hypercholesterolemic	Resveratrol	100	(11 weeks)	SBP		Yes
Rush et al.	2007	Rat	SH	Resveratrol	0.488–4.48 (mg/l)	(4 weeks)	BP and CBF		Yes
Soylemez et al.	2009	Rat	None	Resveratrol	50 (mg/l)	(3 weeks)	SBP	Yes	
Thandapily et al.	2010	Rat	SH	Resveratrol	2.5	(10 weeks)	EVVR	No	No
Toth et al.	2014	Mouse	Ageing	Resveratrol	200	(10 days)	BP	No	Yes
Toth et al.	2015	Mouse	Ageing + Hypertensive	Resveratrol	200	(10 days)	FMD		No
Ungvari et al.	2010	Mouse	Transgenic + HFVD	Resveratrol	2.4 (g/kg chow)	(16 weeks)	BP and EVVR		Yes
Wang et al.	2018	Mouse	T2D	Resveratrol	10	(4–24 weeks)	SBP	No	Yes

Type 2 diabetes (T2D), high fructose vascular dysfunction (FVD), high fat diet (HFD), spontaneously hypertensive (SH), L-NAME induced vascular dysfunction (L-NAME), ex vivo vascular responsiveness (EVVR), systolic blood pressure (SBP), blood pressure (BP), flow mediated dilation (FMD), cerebrovascular blood flow (CBF)

Average intervention dosages can be seen in Table 3 and where possible doses were converted to mg/kg of Body Weight. Mean and median doses were then used to predict optimal doses for human studies, based on established guidelines for conversion [97, 98]. This was carried out for resveratrol interventions in rat and mouse studies, as only these data contained a large enough number of studies for accurate average and conversion calculations. The mean dose of resveratrol in rats was 53.6 ± 156.8 mg/kg (range 1–800 mg/kg), with a median dose of 10 mg/kg, which upon conversion produced an estimated human dose of 8.6 and 1.6 mg/kg for mean and median doses, respectively. In mice, the mean dose was 148 + 135.2 mg/kg (range 10–320), with a median dose of 200 mg/kg, which results in an estimated human dose of 12.0 mg/kg or 11.0 mg/kg for mean and median doses, respectively.

## Human studies

### Study characteristics

Following full-text screening, 42 human studies were identified and included in full data extraction; during which 2 further studies were excluded due to poor study design or a lack of usable primary outcome data [99, 100]. Of the remaining 40 studies, all were randomised, placebo-control studies, with 14 of these of a cross-over design [16–19, 27, 29–31, 101–106] and 26 of which were parallel arm studies [28, 101, 107–130]. Five studies presented results missing primary outcome data for either pre- or post-trial measures and were contacted to request full details, with data being added to extracted data if provided [107, 111, 120, 131, 132]. In addition, two pairs of studies were found to be using data collected within an identical study cohort, consequentially the data were combined into a single entry in the final dataset [29, 122, 133, 123].

Of the included studies, the majority included a disease/at-risk cohort, the most common of which were type II diabetes (*n* = 9), obesity (*n* = 5) and metabolic syndrome (*n* = 4), while six studies investigated only healthy controls. The cohort age ranged from 21 to 65 years (mean = 53.5 years) and studies included both mixed and single sex cohorts (mean = 39.4% female), with an average BMI of 28.5 kg/m^2^ across the full data set. The majority of studies used resveratrol as the supplement intervention (*n* = 25), while only one other study looked at a single RWP in isolation (pterostilbene) [134]. All remaining studies investigated the effects of red wine extract, grape extract, grape seed extract or some modification or combination of these. Five of the included studies investigated acute effects [101, 106, 110, 122, 132], whereas the majority looked at chronic supplementation, of which the most common length was 4 weeks (range = 2—52 weeks) [16–19, 27–31, 103–105, 109, 112–114, 116, 118, 119, 121, 124, 126–130, 134, 135]. To discern the potential differences between acute and chronic effects of RWP, these studies were separated for the purpose of analysis. In acute studies, blood pressure was the only outcome measure that could be carried forward for quantitative analysis, due to low numbers of included studies for other vascular measures (FMD, transcranial doppler ultrasound (TCD), etc.), as well as the wide variation in study design used (e.g., supplement used, dose timing, measurement timing, etc.). Quantitative analysis was carried out on chronic studies presenting blood pressure and/or FMD measures, providing full pre- vs. post-supplementation data were available for both control and supplement study arms.

Quality scores revealed a wide range of study quality: despite all studies being randomised, placebo-controlled studies, only ~ half reported details of the randomisation (51%), concealment (54%) and blinding method used (51%). All studies reported all pre-specified outcome measures, 57% reported dropout rates and 86% reported funding sources and potential conflicts of interest. Overall the mean quality score was 4.4 out of a potential total of 7. The full details of individual scores can be found in Table 4.

**Table 4:** Quality assessment scoring for all human studies included in quantitative data synthesis and analysis, based on the criteria set out in the Cochran Handbook

		Randomisation	Allocation concealment	Blinding	Blinding-outcome	Incomplete data	Selective reporting	Funding	Overall
Barona et al.	2012	0	0	0	0	1	1	0	2
Bhatt et al.	2012	1	−1	−1	−1	1	1	1	1
Bo et al.	2016	1	1	1	1	1	1	1	7
Bo et al.	2013	1	1	1	1	1	1	0	6
Botden et al.	2012	1	1	1	1	1	1	1	7
Draijer et al.	2015	0	0	1	1	1	1	1	5
Evans et al.	2017	1	1	1	1	1	1	1	7
Faghihzadeh et al.	2015	1	1	1	1	1	1	1	7
Fodor et al.	2018	0	1	0	0	0	1	0	2
Fujitaka et al.	2011	0	0	−1	−1	0	1	1	0
Heeboll et al.	2016	0	0	0	0	1	1	1	3
Imamura et al.	2017	0	0	1	1	1	1	1	5
Khodabandehloo et al.	2018	1	1	1	1	1	1	1	7
Kjaer et al.	2017	1	1	1	1	1	1	1	7
Lekakis et al.	2005	0	0	0	0	0	1	0	1
Marques et al.	2018	0	0	0	0	0	1	1	2
Mellen et al.	2010	0	0	1	1	0	1	1	4
Movahed et al.	2013	1	1	1	1	1	1	1	7
Perez-Jiminex et al.	2008	0	0	0	0	0	1	1	2
Ras et al.	2013	0	0	1	1	1	1	1	5
Riche et al.	2014	1	1	1	1	1	1	1	7
Seyyedebrahimi et al.	2018	1	1	1	1	1	1	1	7
Sano et al.	2007	1	1	0	0	1	1	0	4
Sivaprakasapillai et al.	2009	0	0	0	0	0	1	1	2
Timmers et al.	2016	1	1	0	0	0	1	1	4
Timmers et al.	2011	0	0	0	0	0	1	1	2
Tome-Carneiro et al.	2013	0	0	0	0	0	1	1	2
van der Made et al.	2017	0	0	0	0	0	1	1	2
van Mierlo et al.	2010	0	0	1	1	1	1	1	5
Wightman et al.	2014	1	1	1	1	0	1	1	6
Wong et al.	2011	1	1	1	1	0	1	1	6
Wong et al.	2016	1	1	1	1	0	1	1	6
Wong et al.	2013	1	1	1	1	1	1	1	7
Xue et al.	2016	1	1	0	0	0	1	1	4
Yoshino et al.	2012	1	1	0	0	1	1	1	5

### Effect of RWP on blood pressure in humans

Thirty studies reported measures of blood pressure, of which two were acute supplement studies and 28 were chronic (see Tables 5 and 6). Marques et al. [106] showed no significant effect in systolic or diastolic blood pressure following acute administration of grape extract (600 mg). Wightman et al. [101] also found no significant change in blood pressure with resveratrol alone (250 mg) or with resveratrol combined with piperine (250 mg + 20 mg). The combined effects of the three cohorts reported from these two studies showed an estimated mean difference of − 0.6 mmHg [− 2.4, 1.3] and 2.0 mmHg [− 0.4, 4.4] for systolic and diastolic blood pressure, respectively, neither of which was significant and both of which showed high levels of heterogeneity (*p* = 0.547; *I*^2^ = 87.53%, and *p* = 0.108; *I*^2^ = 94.08%, respectively).

**Table 5:** Study characteristics of acute and chronic human studies assessing the impact of red wine polyphenols on systolic and diastolic blood pressure

Author	Year	Study design	Health status	Number of participants	Mean age (years)	Sex (%F)	BMI (kg/m^2^)	Duration (days)	Wash out (days)	Supplement	Dose (mg/day)	Systolic blood pressure (mmHg)		Diastolic blood pressure (mmHg)	
												Control baseline/final (mean ± SD)	Supplement baseline/final (mean ± SD)	Control baseline/final (mean ± SD)	Supplement baseline/final (mean ± SD)
Bhatt et al.	2012	P	T2DM	Control: 29 Supp: 28	57.2	46.9	24.8	90	NA	RSV	250	134.5 ± 14.6/142.3 ± 13	139.7 ± 16.1/127.9 ± 15.4	78.6 ± 10.9/85.7 ± 9.1	81.4 ± 9.6/79.3 ± 9.7
Botden et al.	2012a	X	HT	Control: 61 Supp: 61	61	24.6	27	28	7	Provinol	280	145 ± 12/143 ± 2	145 ± 12/143 ± 2	86 ± 8/83 ± 1	86 ± 8/84 ± 1
	2012b	X	HT	Control: 61 Supp: 61	61	24.6	27	28	7	Provinol	560	145 ± 12/143 ± 2	145 ± 12/142 ± 2	86 ± 8/83 ± 1	86 ± 8/83 ± 1
Draijer et al.	2015a	X	HT	Control: 28 Supp:28	57.6	45	26.3	28	0	GE + GSE	550	139.9 ± 12.2/138.9 ± 1.3	139.9 ± 2.2/135.9 ± 1.3	84.8 ± 8.3/86.6 ± 1.2	84.8 ± 8.3/84.7 ± 0.8
	2015b	X	HT	Control: 28 Supp: 29	57.6	45	26.3	28	0	GE	800	139.9 ± 12.2/132.1 ± 1.4	139.9 ± 12.2/131.8 ± 1.3	84.8 ± 8.3/79.6 ± 0.6	84.8 ± 8.3/79.0 ± 0.6
Faghihzadeh et al.	2015	P	NAFLD	Control: 25 Supp: 25	45.2	30	28.6	84	NA	RSV	500	116.4 ± 14.0/112.9 ± 14.5	119.0 ± 13.8/104.8 ± 12.4	78.0 ± 8.4/74.4 ± 8.6	79.7 ± 8.4/72.8 ± 9.9
Fodor et al.	2018a	P	S	Control: 92 Supp: 81	64.9	39.9	29.7	364	NA	RSV	100	148.4 ± 15.2/145.3 ± 15.3	148.0 ± 15.3/139.9 ± 14.8	87.6 ± 11.3/85.7 ± 11.1	88.3 ± 10.7/84.3 ± 10.2
	2018b	P	S	Control: 92 Supp: 55	64.7	39.2	29.7	364	NA	RSV	200	148.4 ± 15.2/145.3 ± 15.3	149.2 ± 15.1/139.4 ± 14.8	87.6 ± 11.3/85.7 ± 11.1	88.5 ± 11.1/84.1 ± 10.8
Fujitaka et al.	2011a	P	MS	Control: 17 Supp: 17	63	29.4	26.1	90	NA	RSV	100	131 ± 15/129 ± 13	129 ± 14/127 ± 14	74 ± 12/73 ± 14	73 ± 6/76 ± 10
	2011b	P	MS	Control: 17 Supp: 17	62	23.5	27.9	90	NA	RSV	100	131 ± 15/129 ± 13	129 ± 13/133 ± 8	74 ± 12/73 ± 14	73 ± 14/76 ± 15
Heeboll et al.	2016	P	NAFLD	Control: 13 Supp: 13	43.4	34.6	32.1	180	NA	RSV	500	136 ± 15/140 ± 13	142 ± 15/137 ± 16	79 ± 8/84 ± 9	89 ± 8/85 ± 10
Immamura et al.	2017	P	T2DM	Control: 25 Supp: 25	57.8	48	25.1	84	NA	RSV	100	137.1 ± 25.0/133.2 ± 26.5	137.1 ± 18.7/131.6 ± 16.5	80.8 ± 11.5/79.9 ± 11.5	82.0 ± 9.5/80.5 ± 11.2
Khodabandehloo et al.	2018	P	T2DM	Control: 20 Supp: 25	58.9	49	29.6	160	NA	RSV	800	129.2 ± 15.9/126.8 ± 8.2	126.9 ± 9.2/116.9 ± 8.4	74.7 ± 8.3/77.0 ± 8.5	76.8 ± 8.1/70.7 ± 8.2
Kjaer et al.	2017a	P	MS	Control: 24 Supp: 21	48.5	M	33.8	126	NA	RSV	150	150.0 ± 3.4/142.0 ± 2.5	140.0 ± 2.3/145.0 ± 2.6	91.3 ± 2.1/86.0 ± 1.3	86.9 ± 1.5/87.7 ± 1.4
	2017b	P	MS	Control: 24 Supp: 21	49.9	M	34	126	NA	RSV	1000	150.0 ± 3.4/142.0 ± 2.5	146.0 ± 2.3/140.0 ± 2.6	91.3 ± 2.1/86.0 ± 1.3	89.3 ± 1.7/87.8 ± 1.4
Marques et al.	2018	X	ED	Control: 24 Supp: 24	54	58	30	0	7	RSV	300	139 ± 1/141 ± 3	142 ± 2/145 ± 3	87 ± 1/88 ± 2	87 ± 2/88 ± 3
Mellen et al.	2010	X	Pre-MS	Control: 50 Supp: 50	52.1	50	29.8	28	28	RSV	1300	124.6 ± 1.8/123.2 ± 1.0	122.4 ± 1.6/125.2 ± 2.0	75.3 ± 1.2/72.8 ± 1.1	72.8 ± 1.2/73.2 ± 1.3
Movahed et al.	2013	P	T2DM	Control: 33 Supp: 33	52.1	50	27.4	45	NA	RSV	1000	129.3 ± 15.2/130.7 ± 13.2	129.0 ± 14.9/121.5 ± 10.3	78.6 ± 15.4/81.6 ± 5.8	78.6 ± 15.4/78.5 ± 6.4
Perez-Jiminez et al.	2008	P	HC	Control: 9 Supp: 34	35.1	60.1	24.4	126	NA	GADF	7500	121.5 ± 14.0/113.7 ± 9.4	126.5 ± 22.1/118.0 ± 19.6	71.4 ± 14.4/71.3 ± 8.7	78.2 ± 11.7/74.4 ± 12.1
Ras et al.	2013	P	HT	Control:35 Supp: 34	63.7	45.7	25.5	56	NA	GSE	300	135.7 ± 1.7/132.5 ± 1.7	135.8 ± 1.9/130.3 ± 1.7	81.1 ± 1.2/80.0 ± 1.1	81.9 ± 1.5/79.1 ± 1.3
Sano et al.	2007a	P	Healthy	Control: 20 Supp: 21	52.1	51.2	24.3	84	NA	GSE	200	122.7 ± 4.4/127.8 ± 4.8	126.4 ± 3.0/129.2 ± 3.2	77.1 ± 2.6/81.1 ± 2.8	77.9 ± 1.7/79.6 ± 2.0
	2007b	P	Healthy	Control: 20 Supp: 21	52.1	51.7	24.3	84	NA	GSE	400	122.7 ± 4.4/127.8 ± 4.8	126.2 ± 4.0/127.7 ± 3.0	77.1 ± 2.6/81.1 ± 2.8	78.0 ± 2.5/79.5 ± 2.0
Seyyedebrahimi et al.	2018	P	T1DM	Control: 18 Supp: 23	56.8	53.9	28.9	60	NA	RSV	800	129.2 ± 18.2/127.6 ± 12.3	130.0 ± 15.9/113.3 ± 15.9	76.7 ± 8.1/77.0 ± 8.1	76.7 ± 7.9. 72.3 ± 4.1
Sivaprakasapillai et al.	2009a	P	MS	Control: 9 Supp: 9	45.5	61.1	36	28	NA	GSE	150	123 ± 4/121 ± 4	134 ± 5/123 ± 4	74 ± 4/70 ± 4	83 ± 3/77 ± 2
	2009b	P	MS	Control: 9 Supp: 9	46.5	61.1	36.5	28	NA	GSE	300	123 ± 4/121 ± 4	127 ± 4/116 ± 3	74 ± 4/70 ± 4	78 ± 3/71 ± 3
Timmers et al.	2011	X	O	Control: 11 Supp: 11	64	M	30.5	30	30	RSV	150	131.0 ± 3.1/130.5 ± 2.7	132.0 ± 3.0/124.7 ± 3.1	82.0 ± 2.5/81.6 ± 2.8	83.0 ± 0.2/80.0 ± 2.9
Timmers et al.	2016	X	T2DM	Control: 17 Supp: 17	52.5	M	31.5	30	28	RSV	150	142.0 ± 3.9/141.0 ± 2.8	139.0 ± 4.2/138.0 ± 2.9	87.0 ± 2.7/86.0 ± 1.9	85.0 ± 2.7/86.0 ± 1.9
Tome-Carneiro et al.	2013	P	T2DM	Control: 9 Supp: 13	58.5	M	31.4	364	NA	GE	700	129 ± 21/139 ± 23	129 ± 20/137 ± 24	74 ± 13/77 ± 9	73 ± 9/74 ± 11
Van der Made et al.	2017	X	O	Control: 45 Supp: 45	60	44.4	28.8	28	28	RSV	150	136 ± 17/130 ± 18	136 ± 17/132 ± 17	88 ± 9/84 ± 9	88 ± 9/96 ± 9
Van Mierlo et al.	2010a	X	Healthy	Control: 35 Supp: 35	31.4	M	23.2	14	7	GE	1405	123.0 ± 10.5/118.6 ± 1.2	123.0 ± 10.5/121.7 ± 14.5	72.6 ± 9.2/70.8 ± 1.0	72.6 ± 92/71.0 ± 12.4
	2010b	X	Healthy	Control: 35 Supp: 35	31.4	M	23.2	14	7	GSE	2547	123.0 ± 10.5/118.6 ± 1.2	123.0 ± 10.5/122.4 ± 14.5	72.6 ± 9.2/70.8 ± 1.0	72.6 ± 9.2/72.0 ± 11.7
Wightman et al.	2014a	X	Healthy	Control: 23 Supp: 23	21	82.6	24.2	0	2	RSV	250	113.2 ± 11.1/116.6 ± 7.2	112.0 ± 9.5/114.4 ± 6	76.9 ± 11.9/77.6 ± 6.0	75.7 ± 8.0/78.2 ± 3.1
	2014b	X	Healthy	Control: 23 Supp: 23	21	82.6	24.2	0	2	RSV + P	270	113.2 ± 11.1/116.6 ± 7.2	114.2 ± 9.5/115.6 ± 6.0	76.9 ± 11.9/77.6 ± 6.0	75.1 ± 8/79.9 ± 3.1
Wong et al.	2013	X	O	Control: 28 Supp: 28	61	57.1	33.3	42	0	RSV	75	127.4 ± 2.4/128.8 ± 2.8	127.4 ± 2.4/127.6 ± 2.9	73.3 ± 1.3/74.4 ± 1.7	73.3 ± 1.3/74.2 ± 1.4
Xue et al.	2016	X	O	Control: 29 Supp: 29	45	72.4	34.1	56	42	RSV + HESP	210	131.0 ± 2.0/132.0 ± 3.0	131.0 ± 3.0/133.0 ± 3.0	81.7 ± 1.9/82.9 ± 2.3	83.4 ± 2.1/83.3 ± 2.3
Yoshino et al.	2012	P	Healthy	Control: 15 Supp: 16	59	F	24.3	84	NA	RSV	75	123 ± 15/121 ± 14	118 ± 16/119 ± 16	65 ± 10/63 ± 9	67 ± 11/72 ± 10

*X* cross-over, *P* parallel arm, *T2DM* type 2 diabetes melliutus, *HT* hypertension, *NAFLD* non-alcoholic fatty liver disease *S* stroke, *MS* metabolic syndrome, *ED* endothelial dysfunction, *O* Obese, *RSV* resveratrol, *GE* grape extract, *GSE* grape seed extract, *GADF* grape antioxidant dietary fibre, *P* Piperine, *HESP* hesperetin

**Table 6:** Study characteristics of acute and chronic human studies assessing the impact of red wine polyphenols on flow mediated dilation measures

Author	Year	Study design	Health status	Number of participants	Mean age (years)	Sex (% F)	BMI (kg/m^2^)	Duration (days)	Wash out (days)	Supplement	Dose (mg/day)	Flow mediated dilation (∆ %)	
												Control baseline/final (mean ± SD)	Supplement baseline/final (mean ± SD)
Lekakis et al.	2005	P	CHD	Control: 15 Supp: 15	61.0	M	28.0	0	NA	GE	600	2.75 ± 1.85/2.64 ± 1.8	2.6 ± 1.5/4.52 ± 1.34
Marques et al.	2018	X	ED	Control: 24 Supp: 24	54	58	30	0	7	RSV	300	4.1 ± 0.9/2.64 ± 1.8	2.6 ± 1.5/4.52 ± 1.34
Mellen et al.	2010	X	Pre-MS	Control: 50 Supp: 50	52.1	50	29.8	28	28	RSV	1300	5.27 ± 0.42/5.18 ± 0.38	5.22 ± 0.32/4.57 ± 0.32
Van Mierlo et al.	2010a	X	Healthy	Control: 35 Supp: 35	31.4	M	23.2	14	7	GE	1405	5.6 ± 3.6/3.9 ± 2.96	5.6 ± 3.6/3.5 ± 5.66
	2010b	X	Healthy	Control: 35 Supp: 35	31.4	M	23.2	14	7	GSE	2547	5.6 ± 3.6/3.9 ± 2.96	5.6 ± 3.6/4.1 ± 5.66
Wong et al.	2011a	X	O	Control: NA Supp: 18	55	27.8	28.7	0	7	RSV	30	NA	4.06 ± 3.39/6.56 ± 4.76
	2011b	X	O	Control: NA Supp: 18	55	27.8	28.7	0	7	RSV	90	NA	4.06 ± 3.39/6.45 ± 3.61
	2011c	X	O	Control: NA Supp: 18	55	27.8	28.7	0	7	RSV	270	NA	4.06 ± 3.39/7.73 ± 6.34
Wong et al.	2013	X	O	Control: 28 Supp: 28	61	57.1	33.3	42	0	RSV	75	5.83 ± 0.68/5.48 ± 0.69	7.21 ± 0.51/7.42 ± 0.62
Xue et al.	2016	X	O	Control: 29 Supp: 29	45	72.4	34.1	56	42	RSV + HESP	210	0.247 ± 0.327/0.267 ± 0.312	0.207 ± 0.195/0.163 ± 0.195

Xue et al., 2016 results are change in diameter not percentage change

*X* cross-over, *P* parallel arm, *CHD* coronary heart disease, *MS* metabolic syndrome, *ED* endothelial dysfunction, *O* obese, *RSV* resveratrol, *GE* grape extract, *GSE* grape seed extract, *GADF* grape antioxidant dietary fibre, *HESP* hesperetin

Chronic supplementation with a range of RWP resulted in significant changes for measures of systolic (SBP) and diastolic blood pressure (DBP), in 14 and 12 of the included studies, respectively (Table 5). In one study [18], a significant change was seen in the placebo group for DBP, while no significant change was seen in participants supplemented with Muscadine grape seed extract. Of the 28 studies reporting a blood pressure measure, 24 reported pre- and post-trial means for both placebo and intervention groups and were included in quantitative analysis (33 datasets).The remaining 4 studies were not included due to incomplete data (either pre- or post-intervention values not given) for the placebo group [105, 115, 124] or incomplete data in both intervention and placebo groups [16]. Quantitative analysis of pooled data on blood pressure was carried out in 25 studies [17–19, 27–31, 103, 104, 108, 109, 112–114, 116–119, 121, 126–130], with a total of 33 datasets and standardised mean differences calculated.

### Systolic blood pressure

**Fig. 2:**
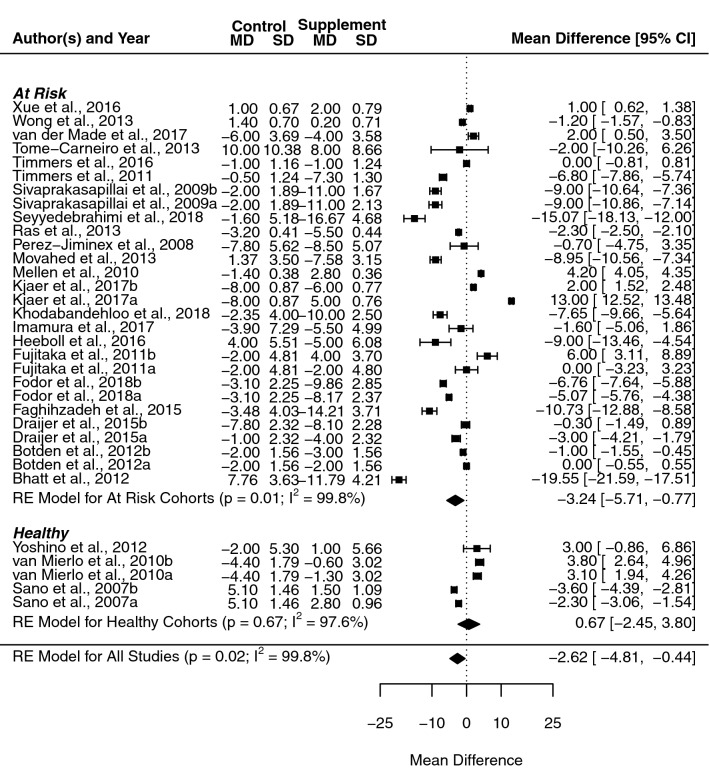
Forest plot showing mean difference and 95% confidence intervals for the impact of chronic red wine polyphenol supplementation compared to placebo-controls on systolic blood pressure in humans, with subgroup analysis based on the health status of the included cohort. Horizontal lines indicate the 95% confidence interval. Shaded diamond shows the calculated subgroup and overall mean difference alongside the results of the random effect model for each group

**Fig. 3:**
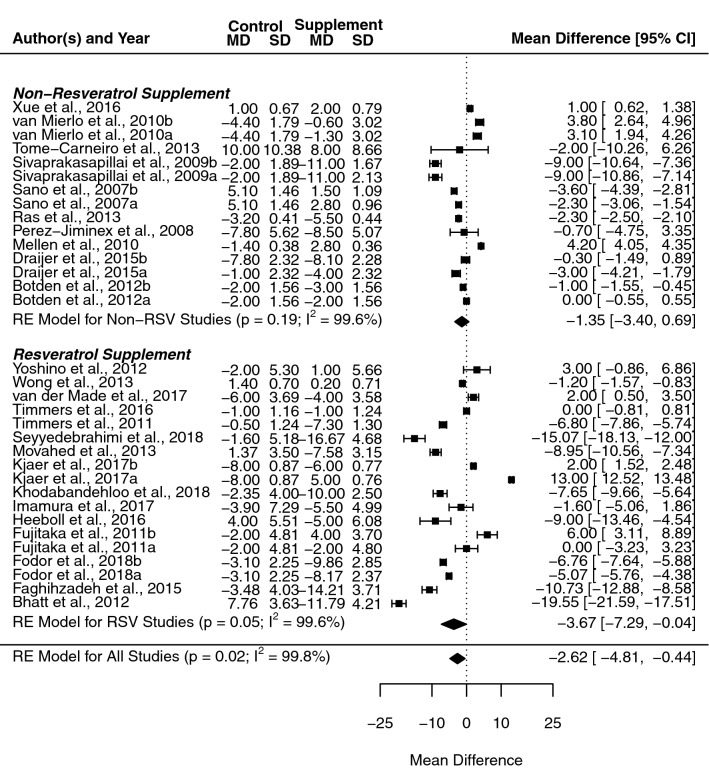
Forest plot showing mean difference and 95% confidence intervals for the impact of chronic red wine polyphenol supplementation compared to placebo-controls on systolic blood pressure in humans, with subgroup analysis comparing studies administering resveratrol compared to all other red wine polyphenols. Horizontal lines indicate the 95% confidence interval. Shaded diamond shows the calculated subgroup and overall mean difference alongside the results of the random effect model for each group

The overall mean difference following RWP supplementation across all included studies was significant for SBP (− 2.6 mmHg, [− 4.8, − 0.4], *p* = 0.010, *I*^2^ = 99.77%). Subgroup analysis revealed a divergence in effect estimates when healthy and at-risk populations were separated (Fig. 2), with a loss of the significant effect in healthy cohorts (0.7 mmHg, [− 2.5, 3.8], *p* = 0.673), while a clear significant effect was seen in at-risk populations (− 3.2 mmHg, [− 5.7, − 0.8], *p* = 0.010). No notable difference was seen in heterogeneity in either subgroup (*I*^2^ = 97.56% and *I*^2^ = 99.81%, respectively). A sufficient number of studies allowed for the separation of resveratrol trials from the wider pool of studies, with separate analysis of resveratrol and all other studies (i.e., non-resveratrol supplement; see Fig. 3). This subgrouping resulted in the maintenance of a significant mean difference in resveratrol studies (− 3.7 mmHg, [− 7.3, − 0.0], *p* = 0.047), but not in the non-resveratrol supplement groups (− 1.4 mmHg, [− 3.4, 0.7], *p* = 0.194); once again subgrouping had no significant effect on heterogeneity in either group (*I*^2^ = 99.63% and *I*^2^ = 99.61%, respectively).

Mixed effect modelling was used to evaluate the collective effects of cohort characteristics (health status, age, sex and BMI), as well as the study design used (parallel or cross-over trials and BP measurement method) and showed that these moderators accounted for 53.7% of heterogeneity, with an omnibus test showing a significant effect for the included moderators (*p* < 0.001). More specifically, health status and measurement method independently accounted for a significant proportion of the heterogeneity (both *p* < 0.001), while study design bordered on significance (*p* = 0.051).

Additional mixed effect models were used to test the potential collective effects of the type, dose and duration of supplementation interventions, as well as the individual effect of medicine status for the at-risk cohort. Neither of these models showed a significant moderator effect (*p* = 0.99 and *p* = 0.127, respectively), although controlling for medication status accounted for 7.7% of heterogeneity in at-risk cohort studies.

### Diastolic blood pressure

**Fig. 4:**
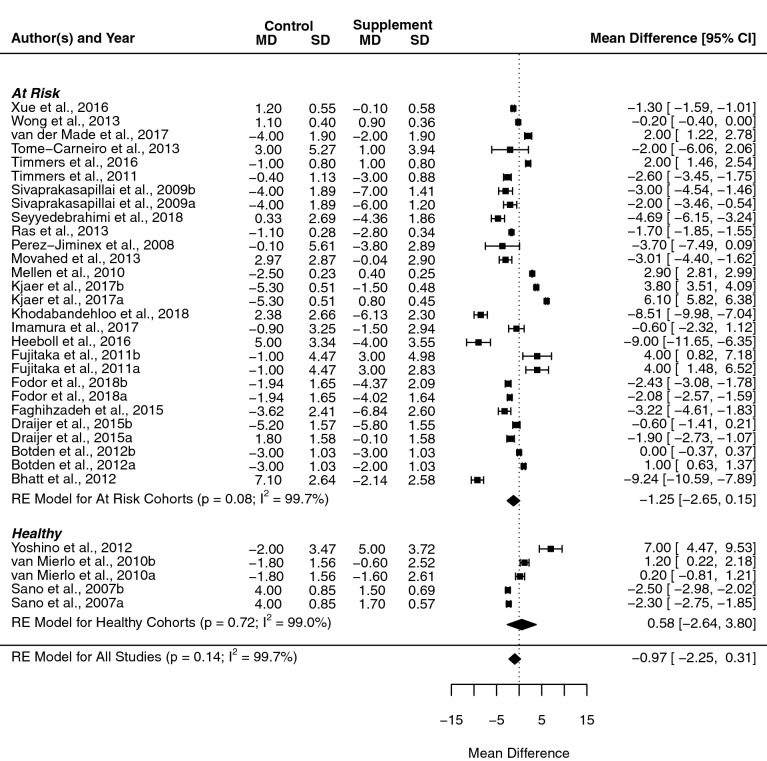
Forest plot showing mean difference and 95% confidence intervals for the impact of chronic red wine polyphenol supplementation compared to placebo-controls on diastolic blood pressure in humans, with subgroup analysis based on the health status of the included cohort. Horizontal lines indicate the 95% confidence interval; shaded diamond shows the calculated subgroup and overall mean difference alongside the results of the random effect model for each group

No significant effects were found in DBP when considering overall effect estimate (− 1.0 mmHg, [− 2.2, 0.3], *p* = 0.139, *I*^2^ = 99.7%), or when subgroup analysis was carried out on resveratrol studies in isolation (− 0.9 mmHg, [− 3.2, 1.3]; *p* = 0.417, *I*^2^ = 99.6%). Subgroup analysis based on health status showed no significance in healthy or at-risk cohorts (Fig. 4). Specifically, there were non-significant changes in healthy individuals (0.6 mmHg, [− 2.6, 3.8], *p* = 0.725, *I*^2^ = 99.0%) and a relative decrease in at-risk cohorts (− 1.2 mmHg, [− 2.6, 0.2], *p* = 0.08, *I*^2^ = 99.7%).

Mixed effect models were used to analyse the same moderators as were investigated in SBP and showed similar results. The cohort-study design model accounted for 40.0% of heterogeneity and this was found to be significant, both overall (*p* < 0.001) and for the health status, BP measurement method and study design moderators individually (*p*  < 0.001, *p*  = 0.032, *p*  = 0.023, respectively). Similarly, the collective supplement moderator model and medicine status model showed no significant impact on heterogeneity (*p*  = 0.998; *p*  = 0.127, respectively), with medicine status accounting for a small amount of heterogeneity (7.2%).

### Sensitivity analysis

**Fig. 5:**
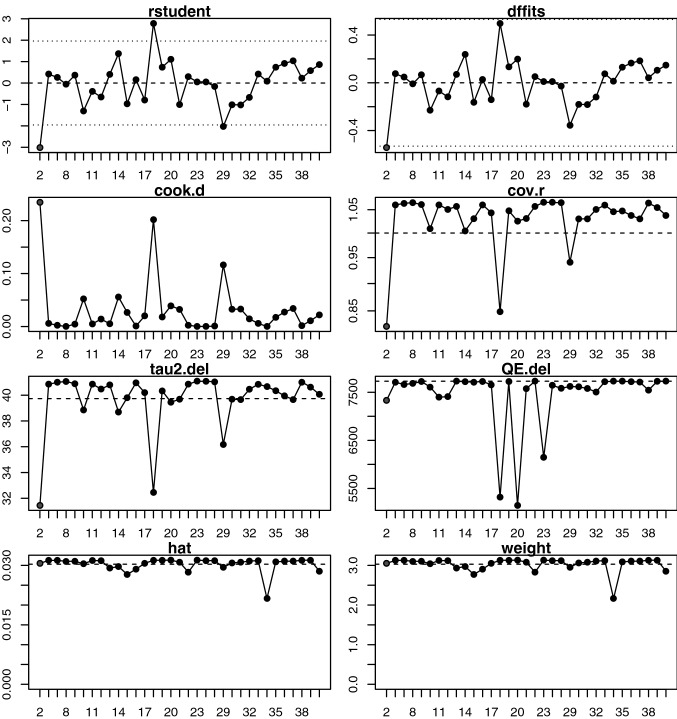
Sensitivity and influence analysis computed using the Metafor meta-analysis package for human studies included in meta-analysis for systolic blood pressure, showing the externally standardised residuals (rstudent), standard deviation fits (DFFIT), Cook’s distances (cook.d), covariance ratios (cov.r), leave-on-out estimates of the amount heterogeneity (tau2.del), leave-one-out values of the test statistics for heterogeneity (QE.del), hat values (hat) and weight weight). Studies presented in red are considered influential and should be inspected on a case-by-case ratio to determine whether they may be considered an outlier

**Fig. 6:**
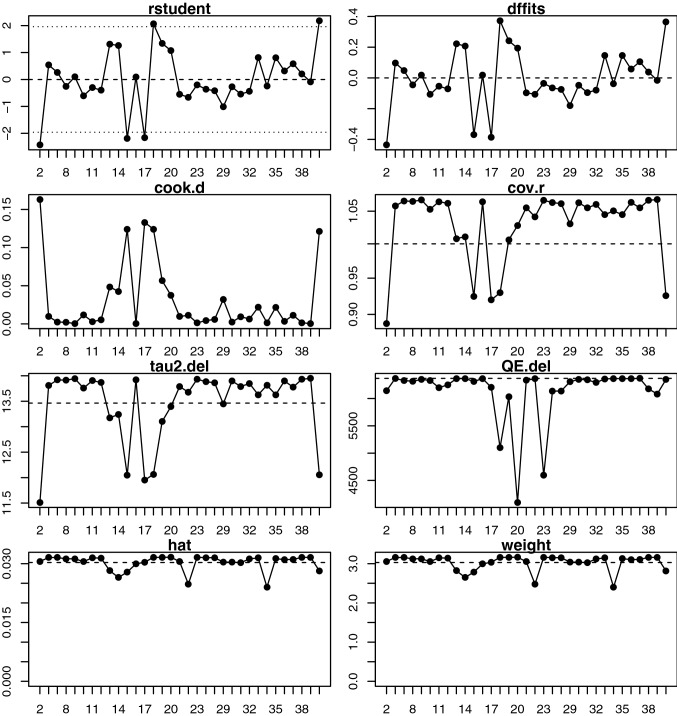
Sensitivity and influence analysis computed using the Metafor meta-analysis package for human studies included in meta-analysis for diastolic blood pressure, showing the externally standardised residuals (rstudent), standard deviation fits (DFFIT), Cook’s distances (cook.d), covariance ratios (cov.r), leave-on-out estimates of the amount heterogeneity (tau2.del), leave-one-out values of the test statistics for heterogeneity (QE.del), hat values (hat) and weight (weight). Studies presented in red are considered influential and should be inspected on a case-by-case ratio to determine whether they may be considered an outlier

Sensitivity analysis identified one outlying study that may have a significant influence on the SBP results [117], with a large residual (*Τ*^2^ = − 3.014) and model fit impact (DFFITS = − 0.542, Cook’s distance = 0.234), although this outlier had little influence on residual covariance or heterogeneity (Fig. 5). Model refitting showed a reduction in the overall effect of supplementation when the outlier was excluded, without a loss of significance or notable change in heterogeneity (− 2.1 mmHg, [− 4.1, − 0.1], *p* = 0.036, *I*^2^ = 99.73). Sensitivity analysis did not reveal any significant outliers for diastolic blood pressure (Fig. 6). Taken in summation, it can be seen that although outlying studies may have been included, these studies did not significantly affect the results of meta-analyses.

### Effect of RWP on vascular function in humans

#### Flow mediated dilation

Peripheral vascular function measures using FMD were reported in 11 studies, of which three were acute interventions [102, 106, 110] and eight were chronic [16, 18, 27, 31, 103, 104, 128, 133]. The full details for each study are presented in Table 6. All three acute FMD studies showed a significant improvement in the FMD response following resveratrol supplementation across a wide range of doses (75–2547 mg/kg/day) [102, 106, 110], as well as supplementation with whole grape extract [110]. Chronic supplementation with RWP had varying effects across the eight studies: with significant increases reported in two studies [16, 128], a significant decrease reported for one [18] and no effect observed in the remaining five studies [27, 31, 103, 104, 133]. Shear rate was also evaluated in three chronic studies with varying results: with no change in two studies [16, 18] and an increase in one study [103].

Quantitative analysis was carried out on the four studies for which full pre-post, placebo-supplement data were available [18, 27, 103, 104]. Random effects modelling estimates showed no significant mean difference between placebo and intervention supplements (− 1.08, [− 4.57, 2.41], *p* = 0.544, *I*^2^ = 99.40%). Health status was evaluated as a potential moderator by mixed effect modelling and showed that this accounted for 72.5% of heterogeneity (*p* = 0.002). Sensitivity analysis revealed one potentially influential outlier [18], which showed a large residual (− 3.844), as well as a notable impact on model fit (DFFITS = − 1.883, Cook’s distance = 0.813) and residual covariance (cov.r = 0.271). Model refitting excluding this outlier resulted in reversal of the effect of supplementation over placebo, but no change in significance (0.526, [− 1.291, 2.343], *p* = 0.570), meaning that although this study impacted the trend seen it did not result in a loss of a significant overall effect.

### Cerebrovascular function

Only two studies were found that measured cerebrovascular function, both of which assessed function using transcranial Doppler ultrasound measures of resting blood velocity in response to resveratrol [122, 125]. Of these, one aimed to determine the acute response to a single dose of resveratrol 75 min prior to assessment [122], whereas the other looked at the response to 12 weeks of supplementation in comparison to a placebo control [125], so comparison of results was not possible. Wong et al. [122] examined acute effects in type 2 diabetics at both high and low doses of resveratrol (75, 150 and 300 mg), reporting significant improvements in middle cerebral artery velocity (MCAv) for all doses, while only low-dose resveratrol resulted in a significant improvement in posterior cerebral artery velocity (PCAv). Moreover, Evans et al. [125] showed that chronic (14 weeks) of resveratrol (150 mg/day) supplementation in a cohort of postmenopausal women significantly increased MCAv response to hypercapnia (i.e., cerebrovascular responsiveness), without a significant increase in resting MCAv.

## Discussion

### General findings

This review aimed to determine the effects of RWP on vascular health, as well as examining the impact of health status and population characteristics on vascular outcomes in both animals and humans. The resulting data showed efficacy within animal models, while human studies displayed larger amounts of variability, which was partly accounted for by known causes of heterogeneity. Overall, the majority of the current literature on RWP was focused on whole grape, seed or skin extracts, or resveratrol in isolation. There was a significant dichotomy in the number of studies between blood pressure and more specific measures of vascular function (e.g., ultrasound measures of flow mediated dilation and cerebral blood flow); likely due to the more time-consuming and technically demanding methods used to assess function. The predominant outcome from the meta-analyses was a significant reduction in SBP within clinical populations, while DBP showed a non-significant reduction following supplementation and compared to placebo cohorts. The following discussion will assess the potential of RWP to have a beneficial effect in vascular health, both generally and in regards to differences seen across different populations and RWP interventions.

### Blood pressure

The findings of the meta-analysis indicated that RWP and isolated grape polyphenol have the capacity to significantly improve SBP beyond the clinical threshold for meaningful effects (− 2 mmHg). The results further indicate that supplementation with resveratrol in isolation may be more effective than supplementation with whole grape or grape seed extract in improving blood pressure. However, this comes with a significant caveat that very high levels of heterogeneity are observed across the studies that have reported SBP as an outcome measure. Although this review did not restrict the type of intervention given, as has been previously described [15, 40, 136], this potential issue was addressed by subgroup analysis of resveratrol alone, alongside meta-regression to investigate supplement type as a moderator; neither of which showed a significant reduction in heterogeneity. Furthermore, despite the greater level of heterogeneity seen in this meta-analysis, the overall findings are in agreement with those reported in previous studies of grape polyphenols and resveratrol [15, 40, 136].

No significant overall effect was found in diastolic blood pressure, although reductions in blood pressure approached significance in the non-healthy sub-group. These findings are consistent with previous reviews in resveratrol [40], but not grape seed extract [15]. The dichotomy between SBP and DBP may be the result of relatively smaller changes in DBP seen in clinical hypertension and the greater the potential impact of heterogeneity between studies as a result of this smaller changes [137]. In addition, within the review by Zhang et al. [15] significance was only maintained in early stage hypertension and “other” clinical studies within subgroup analyses, highlighting the limited consistent findings within this measure. Although both systolic and diastolic blood pressure are indicative of increased morbidity, changes in SBP are considered to be of greater risk in hypertension and CHD [138]. Taken together, these findings indicate that RWP supplementation may be effective at treating hypertension regardless of the lack of consistent responses on DBP.

Importantly, significant heterogeneity was observed across the literature and a number of studies showed an increase in SBP with supplementation, albeit non-significant. Subgroup and meta-regression analysis found no clear pattern with disease type, medication or participant characteristics, indicating that there are unaccounted moderators causing the significant variability in the intervention effects reported. One factor that may have contributed to the large level of heterogeneity may be associated with the variability between placebo and intervention arms at baseline. One likely cause of such variability may related to studies in which blood pressure was not a primary outcome measure; if randomisation was stratified against another outcome measure that could lead to differences in baseline blood pressure between groups. Alternatively, randomisation was not controlled for and this variability is the result of chance, potentially linked to the large number of outcome measures included in some studies. Although BP differences at baseline were not statistically significant in any of the studies considered, they can be considered clinically relevant (> 3 mmHg) in some cases (within the range of the average effect seen under statin treatment [139, 140]). This is a key point that should be taken into consideration in future studies, given that clinically relevant differences in baseline BP measures may increase the risk of type II errors and can be avoided by stratifying randomisation against multiple baseline measures, as opposed to uncontrolled or sex-based stratified randomisation [141].

### RWP and vascular function

The present analysis indicates that RWP supplementation does not result in any significant changes in peripheral vascular function as measured by brachial FMD, with only one study reporting a significant decline in vasodilation following RWP supplementation [18]. Interestingly, this study also showed a significant increase in SBP over 4-weeks of grape seed extract supplementation, indicating that the supplementation had a deleterious effect on the population in this case [18]. Despite the current lack of consistent findings in regards to vascular function, the reported NO-dependent mechanism of action of RWP and more specifically resveratrol [142], would suggest that improvements in measures such as FMD would be expected alongside improvements in blood pressure.

A small number of studies have investigated the specific role of RWP in the cerebrovasculature, with resveratrol supplementation leading to a significant improvement in cerebrovascular function in both studies [122, 125]. The limited number of studies and lack of studies outside of isolated resveratrol supplementation restricts the conclusions that can be drawn regarding cerebrovascular response and warrants further investigation. A direct comparison between the effects of supplementation on the different vascular beds will help to determine whether RWP effects are indeed distinct across vasculatures, or due to limitations of the experimental approaches used to detect the response to RWP supplements.

### Animal study results and transition into humans

Findings from animal studies consistently demonstrated significant improvements in both blood pressure and vascular function in disease models across a wide variety of supplement types and pathologies. In particular, studies into the effects of RWP on vascular responsiveness showed improved vasodilatory response in excised arterial rings in all cohorts, regardless of disease model or species [6, 8, 10, 58, 60, 65, 67, 78, 85, 87–89, 92, 96, 143, 144]. In regards to effects on blood pressure, the large majority of studies showed significant improvements (84%) [6, 7, 9, 57–60, 64, 67, 73, 77–79, 82–86, 88, 93, 95, 144–150], while a smaller number of studies did not show any beneficial response to supplementation. Interestingly, in all studies showing no significant improvement in blood pressure [69, 70, 75, 81, 94], a significant improvement was seen in vascular responsiveness, indicating that supplementation did have an effect within the vasculature despite no significant changes in blood pressure being found. In the three studies involving hypertensive models that saw no significant improvement [69, 81, 94], no consistent pattern was seen in regards to study duration, dosage, supplement type or animal model used, which could explain the differences seen between these studies and the majority of findings.

Consistent effects were not seen in healthy animals, with less than half of studies showing a beneficial change in blood pressure and vascular responsiveness outcomes following supplementation. This indicates that even in the highly controlled environment in which animals are raised, RWP have limited potential to improve vascular health or function above the ‘healthy physiological normal’. This generally agrees with what has been reported in human studies, in which no significant impact was seen across healthy cohorts.

The lack of reproducibility from animal models to humans is well documented and is by no means unique to nutrition research [151]. This is clearly visible within this review, wherein despite similar trends in regards to the benefit in healthy and at-risk populations, the response to RWP in at -risk and disease populations was markedly more consistent in animal models than in human studies. Broadly speaking the potential causes of this variation fall into three categories: (i) issues in effectively transitioning animal-model research into humans; (ii) the condition differences in which studies can and are conducted between animals in captivity and humans; and (iii) the biological differences between animals and humans. One of the most common issues to be considered in study designs when trying to emulate animal model results in humans is that of dosage. Traditionally when transitioning from animal to human models, it was common practice to directly translate dosage in milligrams per kilogram of body mass. However, it has now been shown that due to the higher metabolic rate in smaller animals a more appropriate method is to convert doses based on body surface area (BSA) [97]. Within the studies included in this review, the dose administered varied widely across both human and animal studies, highlighting there is as yet no clear consensus on optimal dosage in either field. When resveratrol doses were compared between rodents and humans, median doses were greater in humans when compared to rats (conversion ratio 1:6.2), but lower than that used in mice (1:12.3) [97, 98]. Given the similarity in results between rats and mice, these results would indicate that either the dosage used in mouse studies is in excess of what is required to see an effect, or that a significant variation in responses is seen between species. If the latter is the case then the same may be true when comparing humans to animal models and future research must establish the dose response curve in humans specifically, rather than relying on dose conversion from animal studies. In addition, it should be noted that future studies in animals need to establish clear optimal doses to exclude variability in dosage as a potential cause for variability in outcome.

Greater variability in outcome measures of human studies is to some degree unavoidable, as replicating the level of control experienced in animal studies is near impossible in a free-living population of humans. Furthermore, the introduction of dietary polyphenols into an animal population can be guaranteed to be novel, as chow diets can be ensured to be free of these compounds. In humans consumption during intervention can be controlled to some extent, however, adherence to polyphenol free conditions is likely to be as problematic as adherence to other dietary interventions [37, 38], while controlling for lifetime consumption is simply not possible. This might result in a fundamental difference in the physiological response in humans and animals, given that rodent models are being exposed to these compounds for the first time, whereas humans are likely to have been previously exposed to them and may have variable responses dependent on their dietary background. In addition, despite the present review including only animal studies in which supplementation was carried out through oral administration, differences in compound delivery still occur: e.g., through oral gavage [60], incorporation into chow [89], or via drinking water [152], which ultimately will cause inter- and intra-subject variability in the dose consumed and its bioavailability.

Furthermore, distinct absorption and metabolism are likely key determinants of the variability in physiological responses to dietary polyphenols across species. Generally, polyphenols have poor bioavailability in their original form and are dependent on the activity of circulating metabolites to elicit beneficial effects [34, 153]. The complex metabolic fate of combined RWP [154] within human populations, alongside the dependence on the lower digestive tract for absorption [34], highlights the potential for variability between bioavailability humans and animal models. Direct comparison of metabolic fate between humans and rodents of the dietary polyphenol epicatechin found in grapes, as well as in high levels in dark chocolate, demonstrated the striking differences that can be found when determining metabolic fate [155]. Furthermore, Ottaviani et al. [155] also highlighted the importance of gut microbiota in defining this fate, as large changes in circulating metabolites occurred following sufficient time for the dose to the colon. Clear differences have also been found in the metabolic fate of resveratrol and pterostilbene (a dimethyl analog of resveratrol), due largely to significant differences in the microbial biotransformation of both polyphenols prior to absorption into the blood stream [156]. Once again indicating that the differences in gut microbiota between species can have a significant impact on the impact of dietary polyphenols.

Although animal models do provide unique opportunities to explore the mechanistic and structural responses to RWP supplementation, the differences between animal models and free living humans dictate that findings from these models cannot readily predict responses in human populations [157]. To maximise the potential for human studies to replicate the results seen in animals, researchers must ensure adherence to both intervention and dietary restrictions are monitored closely and undertaken further work to establish the differences in biological response under supplementation.

### Causes of heterogeneity

The large level of heterogeneity within this meta-analysis was not unexpected, particularly when considering the data as a whole, since multiple types of interventions (pure polyphenols and more complex extracts) were included, as well as various vascular dysfunctions associated with “at-risk” populations. However, meta-regression results based on the moderators identified in previous meta-analyses accounted for just over half of variability between studies, with no significant heterogeneity accounted for by dose and duration of intervention, which have previously been reported as major sources of heterogeneity [15, 40]. Disease characteristics appear to be responsible for the largest proportion of accountable heterogeneity, despite the similarity in causes of vascular endothelial dysfunction between many of the included diseases [158–160].

Given the impact of disease characteristics and the lack of effect when controlling for medication, it may be that there is an unknown interaction between the specific treatments for each condition and RWP supplementation, which could go some way as to explain the lack of significant interaction with medication status. Alternatively, the wide variety of vascular-linked diseases and the wide variations in the symptoms presented by each disease may all alter the response to RWP supplementation. Regardless, it does appear that the response to RWP supplementation is highly variable across different pathologies and this must be taken into account when considering the efficacy for supplementation within a given cohort.

### Whole extracts or isolated components

To the best of our knowledge, this is the first review, that has sought to directly compare the vascular response to whole RWP interventions with pure resveratrol, both of which have been suggested to have vasoprotective properties [23, 24]. The results of this meta-analysis indicated that resveratrol alone produces similar vascular responses to those seen in whole grape and grape seed extract supplements. Furthermore, resveratrol seems to produce more consistent results than those seen in whole extracts, as indicated by the greater effect size and lower number of equivocal findings. There are clear benefits to supplementation with resveratrol in isolation, as it is purified and produced at a given concentration per dose. Whole extracts can vary in the relative content of individual components and optimal dosage will vary dependent on grape variety and extraction process. It should be noted that there are potential issues with resveratrol supplementation, as research has highlighted that although oral absorption is relatively high, the bioavailability of resveratrol in the blood stream was low [161]. Conversely, there are additional stilbene compounds, such as pterostilbene, that exhibit similar effects as resveratrol while displaying improved bioavailability [162].

### Recommendations for future studies

Before recommendations can be made regarding any form of RWP supplement, further research should be conducted to determine the efficacy, optimal dose and minimum duration for that supplement within a target cohort. The studies included in this review demonstrate the lack of a clear consensus on dosage, as even single supplement studies demonstrated a wide dosage range. For example, resveratrol was administered at a minimum dose of 75 mg/day [28, 103] and a maximum of 1000 mg/day [109, 112].

Alongside the need for greater clarity on dose and duration responses, future studies must look to determine the specific interaction of different medications. Indeed, our analysis indicates that only taking into consideration whether a participant cohort is taking medication does not appear to explain the differences in outcomes observed between studies. Future studies should also look to determine the efficacy of other stilbenes and polyphenols in improving vascular function, given that resveratrol is one of many potentially vasoactive components found in red wine and grape extracts. To better determine the effects of RWP in vascular dysfunction, more research that is less reliant on measures of blood pressure alone is needed, as a loss of vascular responsiveness is also an essential component of disease risk and at present there is a notable shortage of studies identifying peripheral and cerebral vascular function changes with RWP supplementation. In addition, Gliemann et al. [163] demonstrated that the adaptation response to regular exercise was blunted with resveratrol supplementation in older men and further studies are essential to determine if this is an issue with resveratrol and RWP supplementation in general, as regular exercise has been consistently shown to improve vascular health and risk factors [164].

### Limitations

In this meta-analysis a large amount of heterogeneity was accounted for as a result of specific data on disease status and disease type. H, detailed meta-regressions were not possible with regards to medication status, as the data available only indicated whether a cohort as a whole was medicated. Due to the large number of health conditions within the included studies and the potential variation in interaction effects with each prescribed medication, it was not possible to determine the impact of specific medicines on the effects of a given supplement and to do so would increase the risk of type I error [51]. In future, reviewers will need to establish a method for addressing or identifying the interaction of specific medications with a given supplement to control for this moderator across a wide range of populations, or alternately determine the interaction effect within a less varied population with a smaller number of potential medications. Secondly, full comorbidity and multimorbidity data were not extracted from included studies, which prevented us from assessing the impact that this had on results and heterogeneity. Future studies will need to determine how this can be done as vascular dysfunction rarely appears in isolation and a number of diseases are characterised by the accumulation of multiple conditions (e.g., metabolic syndrome), making differentiating between primary disease and comorbidity difficult. Finally, due to the low number of studies in peripheral and cerebral vascular function, only basic effect size could be calculated for FMD outcome measures. As such, limited conclusions could be drawn and it was not possible to identify potential moderators with regards to vascular responsiveness measures.

## Conclusion

This review has shown that RWP supplementation has the capacity to improve SBP in human clinical populations, but with no clear response in DBP and vascular function (as measured by brachial FMD). Furthermore, we have shown that pure resveratrol was as effective as whole RWP extract supplementation in improving blood pressure and vascular function. Animal models, most of which were rodent models of disease, have shown a consistent and large response in all markers of blood pressure and in vitro and in vivo vascular function. In comparing human and animal data, although some of the beneficial responses seen in rodent models appear to be carried over into humans, the consistency and magnitude of the changes seen are not emulated and this is likely due to differences in biology, lifestyle and experimental control. Finally, there is significant heterogeneity within the literature as to the efficacy of RWP interventions targeting human vascular health and function, and for this reason future research needs to address the scope of conditions for which RWP are beneficial and the dose and duration required for a given intervention and population. Overall, at present it is not possible to accurately predict the effects of RWP supplements due to the significant levels of heterogeneity between studies. Further research in RWP must focus on how to improve consistency and generalisability of findings through more effective control of confounding factors, such as medication status, diet composition and daily physical activity levels.
